# Lactoferrin and Nanotechnology: The Potential for Cancer Treatment

**DOI:** 10.3390/pharmaceutics15051362

**Published:** 2023-04-28

**Authors:** Tuan Hiep Tran, Phuong Thi Thu Tran, Duy Hieu Truong

**Affiliations:** 1 Faculty of Pharmacy, Phenikaa University, Yen Nghia, Ha Dong, Hanoi 12116, Vietnam; 2 Department of Life Sciences, University of Science and Technology of Hanoi, Vietnam Academy of Science and Technology, 18 Hoang Quoc Viet, Cau Giay, Hanoi 10000, Vietnam; 3 Bac Ly Ward, Dong Hoi City 510000, Vietnam

**Keywords:** lactoferrin, nanoparticles, cancer, targeting, therapy

## Abstract

Lactoferrin (Lf)—a glycoprotein of the transferrin family—has been investigated as a promising molecule with diverse applications, including infection inhibition, anti-inflammation, antioxidant properties and immune modulation. Along with that, Lf was found to inhibit the growth of cancerous tumors. Owing to unique properties such as iron-binding and positive charge, Lf could interrupt the cancer cell membrane or influence the apoptosis pathway. In addition, being a common mammalian excretion, Lf offers is promising in terms of targeting delivery or the diagnosis of cancer. Recently, nanotechnology significantly enhanced the therapeutic index of natural glycoproteins such as Lf. Therefore, in the context of this review, the understanding of Lf is summarized and followed by different strategies of nano-preparation, including inorganic nanoparticles, lipid-based nanoparticles and polymer-based nanoparticles in cancer management. At the end of the study, the potential future applications are discussed to pave the way for translating Lf into actual usage.

## 1. Introduction

Lactoferrin (Lf), a protein with iron-binding ability, was first discovered in mammary secretions in 1939. Lf is a protein with a large molecular size of ~80 KDa consisting of ~700 amino acids stabilized by disulfide bonds [1]. Three different isoforms have been found, including: Lf-α—the iron-binding isoform, while the non-iron binding isoforms (Lf-β and Lf-γ) have ribonuclease activity [2]. By the ability to bind iron, Lf restricts the iron availability to microbes; hence, Lf has a significant impact on the lives of microbes. Moreover, Lf has also been found to influence the innate and adaptive immune systems by which Lf can communicate with most immune cells, such as antigen-presenting cells, granulocytes, natural killers, lymphocytes, macrophages, and neutrophils, and then trigger a down-stream signaling pathway via cytokines. This was described and discussed thoroughly by Sienkiewicz et al. [3].

The relevant biological functions of lactoferrin have been reported, including anticancer, antibacterial, antiviral, antifungal, anti-inflammatory, and immune-regulatory activities [4]. It was shown that alterations of the lactoferrin gene in cells could be associated with an increased incidence of cancer. Exogenous treatment with lactoferrin and its derivatives could efficiently inhibit the growth of tumors and reduce susceptibility to cancer [5]. Several studies have reported that lactoferrin caused cytotoxicity against several cancers via cell membrane disruption, apoptosis induction, and cell cycle arrest. In detail, due to the high pI value (pI~8.0–8.5), in the normal environment, Lf exists as a positively charged molecule that likely has a high affinity to the negative charge of the cancer cell membrane. In addition, the key players of the apoptosis pathway, including caspase 3, caspase 8, Bcl-2, p53, and p21, were found to be modulated toward cancer cell growth inhibition [6,7,8,9,10,11].

The biological activities of Lf are revealed via the interactions between Lf and receptors, including CD14, cytokine receptor 4 (CXCR4), heparan sulfate proteoglycans (HSPGs), intelectin-1 (omentin-1), LDL receptor-related protein-1 (LRP-1/CD91), and Toll-like receptors (TLR2 and TLR4) [2]. By exploiting these interactions, Lf can potentially be utilized for the targeting strategy [12]. Those overexpressed receptors on the cancer cell surface favor the receptor-mediated endocytosis of Lf-coated carriers, leading to the enhancement of substance delivery [13].

In cancer treatment, lactoferrin itself is challenged by its complex structure, which can be figured out with the support of advanced delivery strategies. Nanomedicine—the field that was initiated since the approval of Doxil^®^—is developing quickly for cancer diagnosis and treatment [14]. The nanocarrier systems offered the therapies a variety of advantages, such as (i) enhanced solubility and bioavailability, (ii) enhanced biological stability and retention, and (iii) improved ability to reach the expected sites via active targeting delivery [15]. Bearing these in mind, in this review, we summarized different approaches to use Lf as the targeting ligand to improve the delivery of substances to the cancer sites and as the active agent to manage the growth of tumors. The review is constructed following the types of material-based nanosystems such as inorganic nanoparticles, lipid nanoparticles, polymeric nanoparticles, and drug-Lf conjugates. Future perspectives involving some discussions on the development of Lf could help to bring it into clinical use.

## 2. Nanoparticles with Lactoferrin as a Targeting Moiety

### 2.1. Inorganic Nanoparticles Modified by Lactoferrin

#### 2.1.1. Silica Nanoparticles

Nearly 80% of malignant brain tumors originating in the brain are classified as gliomas, significantly shortening life expectancy more than other types of tumors [16]. Glioblastoma, which is the most prevalent form of glioma, has a very low survival rate. Unfortunately, the blood–brain barrier (BBB) hinders the transport of therapeutic agents into the brain, thereby limiting the effectiveness of many pharmaceuticals that have been proven to be effective for other tumor types [17]. A new delivery platform, Lf-USLPs, has been created by conjugating lactoferrin to ultra-small sized, large-pored silica nanoparticles (USLPs) to deliver doxorubicin (DOX), which cannot penetrate the BBB, for glioblastoma treatment [18]. In vitro tests showed that Lf-USLPs selectively permeated the BBB, effectively internalized into U87 glioblastoma cells, and significantly penetrated U87 tumor spheroids compared to uncoated USLP-NPs. Additionally, the USLP-Lf platform markedly enhanced DOX-induced apoptosis in U87 cells in both 2D and 3D tumor spheroid models. A recent study of the same research group using Lf-USLPs for the delivery of temozolomide (TMZ) also revealed the improved BBB permeation and reduced efflux ratio of TMZ [19]. Moreover, in vitro studies showed that TMZ-loaded Lf-USLPs enhanced apoptosis in U87 and GL261 glioblastoma cell lines compared to pure TMZ.

**Figure 1:**
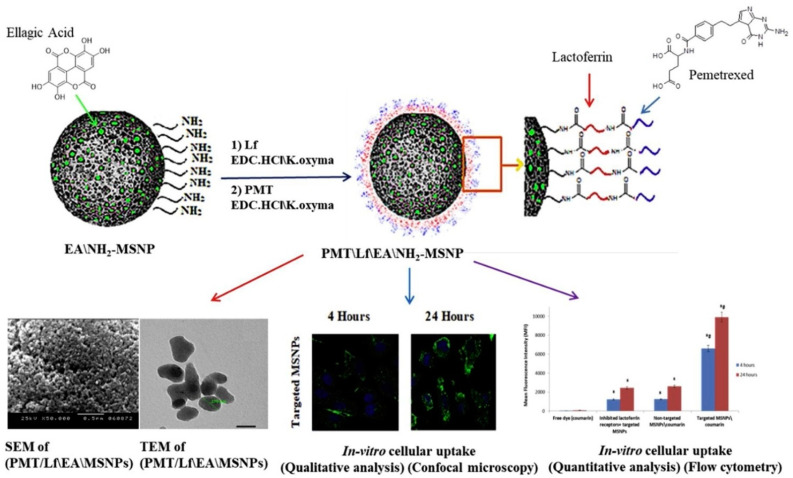
Preparation and characteristics of dual drug-loaded lactoferrin-modified mesoporous silica nanoparticles (MSNPs). EA, Ellagic acid; EDC.HCl, 1-ethyl-3-(3-dimethylamino)propylcarbodiimide hydrochloride; K-Oxyma, Ethyl (hydroxyimino)cyanoacetate potassium salt; Lf: Lactoferrin; PMT, pemetrexed. Reprinted with permission from [23].

As a biocompatible compound group with several advantages, such as high surface area and easy surface functionalization, mesoporous silica nanoparticles (MSNs) have long received much attention in drug delivery and biomedical applications [20,21,22]. An injectable formulation of Lf-conjugated MSNs co-loading pemetrexed (PMT) and ellagic acid (EA) has been shown to increase the cytotoxicity in the MCF-7 breast cancer cell line, resulting from the high local internalization of the conjugated nanoparticles and synergistic effect between the two drugs, compared to the combination of free drugs (Figure 1) [23].

#### 2.1.2. Magnetic Nanoparticles

The unique magnetic properties have made magnetic nanoparticles excellent biomedical materials in disease diagnosis and treatment, such as in magnetic resonance imaging (MRI) and hyperthermia [24,25,26].

In a study, two types of magnetic iron oxide nanoparticles (IONPs), namely maghemite and magnetite NPs, were synthesized and decorated with Lf [27]. These two Lf-modified nanoparticles showed higher cellular internalization into 4T1 breast cancer cells than non-modified nanoparticles, thereby increasing the cytotoxicity in this cell line. Another formulation of lactoferrin–doxorubicin–mesoporous maghemite nanoparticles (Lf–DOX–MMNPs) was designed for the targeted delivery of DOX to treat breast cancer and reduce its metastasis [28]. The results showed that the Lf–DOX–MMNPs not only improved the efficacy of cancer treatment in vitro, but also led to significant tumor growth inhibition in vivo. Notably, the combination therapy of chemotherapy, magnetic field, and photothermal therapy using the Lf–DOX–MMNPs significantly delayed the metastasis of breast tumors.

A targeted delivery system encapsulating DOX was made from graphene oxide (GO) sheets by adding superparamagnetic iron oxide nanoparticles to its surface, followed by conjugation with Lf [29]. Compared to free DOX and unconjugated nanoparticles, Lf-conjugated ones showed better intracellular internalization and higher toxicity to C6 glioma cells. In another study, a dual-targeting magnetic nanocarrier system, known as polydiacetylene nanocarriers (PDNCs), modified with Lf, has been fabricated with micelles-polymerized structures [30]. The results showed that the magnetic Lf-modified PDNCs, with the ability to be tracked via magnetic resonance imaging (MRI) and dual-targeting, were able to improve the transportation across the BBB for glioma tracking and targeting. Furthermore, Lf-coated curcumin (CUR)-loaded PDNCs enhanced the concentration of CUR in the brain compared to the free drug and remarkably inhibited tumors in RG2 cell-bearing rats.

**Figure 2:**
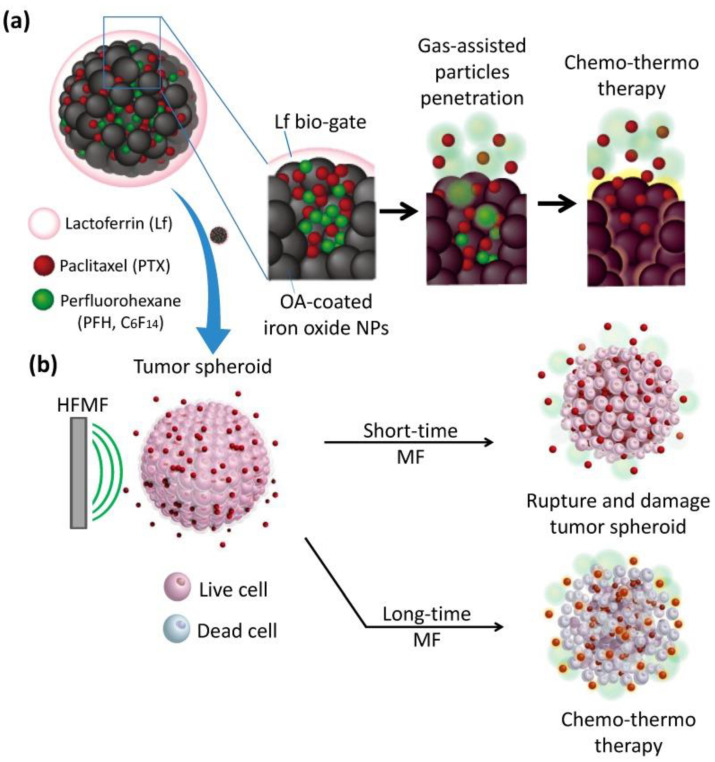
(**a**) Schematic illustration of the lactoferrin-capped mesoporous iron oxide nanoparticle (Lf-MION). The hydrophobic pores of Lf-MION carry both paclitaxel (PTX) and perfluorohexane (PFH), and these cargos can be triggered when treated with a high-frequency magnetic field (MF). (**b**) MF-induced heat gasifies PFH to enhance the penetration and accumulation of Lf-MIONs in a tumor spheroid. Through a combination of thermo-gasification and thermo-chemotherapy, the Lf-MIONs can effectively eradicate the cancer cells in tumor spheroids. Reprinted with permission from [32].

Fang et al. developed nanocarriers with a magnetic core–shell structure composed of Lf attached to double emulsion nanocapsules, which were assembled from iron oxide nanoparticles, polyacrylic acid (PAA), and polyvinyl alcohol (PVA), for the simultaneous delivery of both hydrophobic CUR and hydrophilic DOX to brain tumors [31]. The findings revealed increased cytotoxicity in RG2 glioma cells, and a significant reduction of tumor size compared to free drugs or single drug-loaded nanocarriers in mice bearing RG2 cells under magnetic guidance. In another study, mesoporous iron oxide nanoparticles (MIONs) coated with LF were synthesized with the ability to deliver a high amount of gas-formed perfluorohexane (PFH) and paclitaxel (PTX) (Figure 2) [32]. Under the magnetic field treatment, PFH-assisted gasifying enhanced the penetration of the nanoparticles and drug accumulation inside the tumor, and the higher MION-triggered local temperature facilitated the sustained release of PTX. This phenomenon led to the damage and deformation of tumor spheroids and enhanced cytotoxicity in vitro. Additionally, the tumor growth inhibition in mice bearing an RG2 tumor was significantly increased in the LF-coated dual drug-loaded MIONs compared to all other nanoparticles, signifying the synergistic efficacy of chemotherapy, hyperthermia, and deep tumor penetration.

#### 2.1.3. Other Inorganic Nanoparticles

Despite advances in medical technology, accurately diagnosing and treating glioblastoma remains a challenge [33]. Although near-infrared (NIR) light-triggered fluorescence imaging and photodynamic therapy show promise for the theranostics of glioblastoma, their effectiveness is limited by the presence of the BBB and hypoxia [34,35]. To address these shortcomings, Lv et al. developed a new theranostic nanoagent with a YOF:Nd^3+^ core and a MnO_2_ shell to encapsulate the photosensitizer indocyanine green and glucose oxidase, followed by functionalization with Lf [36]. The excellent fluorescence performance of its core facilitated the highly sensitive diagnosis of orthotopic glioma. The reaction of glucose oxidase and glucose produced glucuronic acid and hydrogen peroxide (H_2_O_2_), achieving starvation therapy. Subsequently, the catalysis of H_2_O_2_ by MnO_2_ to release oxygen improved photodynamic therapy. The synergistic effect of these two therapies significantly inhibited tumor growth in mice bearing GL261 glioma cells.

Recently, Cao et al. developed a synergistic chemo/chemodynamic/photothermal/starvation therapy to deliver TMZ for glioblastoma treatment [37]. In this nanoplatform, TMZ was loaded in hollow mesoporous copper sulfide nanoparticles, which also play a significant role in highly selective photothermal therapy, using hyaluronic acid as a gatekeeper to prevent premature drug leakage and enhance responsive drug release at tumor sites. The nanoplatform was modified with glucose oxidase to self-supply H_2_O_2_ and glucuronic acid for chemodynamic therapy and to consume glucose for starvation therapy. Further modification with Lf achieved active targeting of the nanoplatform for better antitumor efficacy in vitro and in vivo under laser irradiation, verifying the great potential of this multimodal platform in glioblastoma treatment.

### 2.2. Lipid-Based Nanoparticles Modified with Lf

#### 2.2.1. Liposomes

Liposomes are spherical vesicles whose membranes are composed mainly of a variety of phospholipid bilayers [38]. Liposomes have been used as drug carriers in cancer treatment to enhance the stability of the encapsulated materials and protect them from the surrounding environments [38,39,40].

Chen et al. fabricated and evaluated the anti-tumor efficacy of DOX-loaded procationic liposomes using Lf as a targeting ligand for glioma treatment [41,42]. They used the thin-film hydration method to prepare negatively charged liposomes on which positively charged Lf was absorbed. In comparison to uncoated liposomes, the Lf-coated liposomes demonstrated higher anti-glioma efficacy, resulting from enhanced cellular uptake and BBB permeability.

To further enhance brain targeting, docetaxel (DTX)-encapsulated liposomes were co-modified with RGD peptide, which can specifically bind to integrin αvβ3 receptor on glioma cells, and Lf [43]. Co-modification facilitated the penetration into U87 tumor spheroids and distribution in brain orthotopic gliomas. In addition, co-modified DTX-encapsulated liposomes markedly improved the survival in mice bearing U87 MG. Recently, Qi et al. tried to fabricate DTX-loaded liposomes co-modified with muscone, which can facilitate drug permeability across the BBB [44,45], and Lf [46]. The dual-modified liposomes not only enhanced cellular internalization into U87 MG glioma cells and hCMEC/D3 cells but also increased penetration into U87 MG tumor spheroids and the hCMEC/D3 BBB model. Moreover, the dual-modified liposomes demonstrated the highest in vitro and in vivo anticancer efficacy among the four groups tested.

To target hepatocellular carcinoma (HCC) overexpressing Asialoglycoprotein receptors (ASGP-R) [47,48], Lf was covalently coupled to the surfaces of DOX-loaded PEGylated liposomes [49,50]. Compared to PEGylated liposomes, Lf-modified PEGylated liposomes exhibited higher uptake in the ASGP-R-positive cells, leading to enhanced cytotoxicity in vitro. Significantly higher tumor growth inhibition in HepG2 cell-bearing mice confirmed the Lf-modified formulation’s superior anti-tumor activity [50].

Zhang et al. created PEGylated liposomes modified with holo-lactoferrin (holo-Lf) to encapsulate the anticancer drug DOX for combined radiochemotherapy in cancer treatment [51]. Holo-Lf has natural targeting abilities, allowing Lf-modified liposomes to accumulate in cancer cells. The authors also discovered that holo-Lf could catalyze the conversion of hydrogen peroxide (H_2_O_2_) to oxygen, thereby reducing tumor hypoxia, which was confirmed using photoacoustic imaging. Combined radiochemotherapy in mice bearing 4T1 breast cancer cells showed significantly higher tumor inhibition when using DOX-loaded Lf-modified liposomes compared to radiation therapy or DOX-loaded liposomes plus radiation therapy.

**Figure 3:**
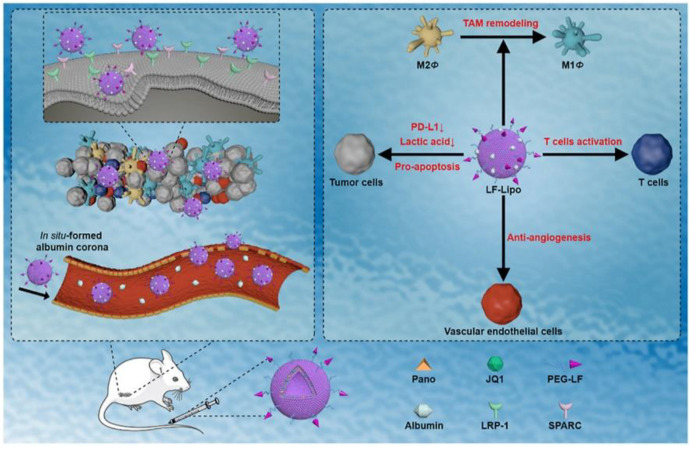
The lactoferrin-modified and albumin-adsorbed liposome for targeting the tumor microenvironment was designed for epigenetic-based combination therapy. This tumor-targeting liposome can activate anti-tumor immunity responses, inhibit aerobic glycolysis and angiogenesis, and restrict tumor growth and metastasis via epigenetic regulation. Reprinted with permission from [54] under a Creative Commons Attribution-NonCommercial-NoDerivatives 4.0 International (CC BY-NC-ND 4.0) License.

The regulation of epigenetics, cancer metabolism, and immunology are closely linked, and a combination of epigenetic therapy and immunotherapy has promising potential for managing cancer [52,53]. Recently, He et al. proposed a novel therapy to remodel the tumor immune microenvironment (TIME) for metastatic colorectal cancer that involves a chemo-free, epigenetic-based combination of panobinostat and JQ1 (Figure 3) [54]. An epigenetic regulator—panobinostat—can promote histone acetylation, hinder tumor cell proliferation, regulate aerobic glycolysis, and reprogram intratumoral immune cells [53,55], while a Bromodomain-containing protein 4 inhibitor—JQ1—reduces Programmed death ligand 1 (PD-L1) expression [56]. The modification of lactoferrin and the adsorption of endogenous albumin have a dual-targeting effect on the receptors of both LRP1 and Secreted protein acidic and rich in cysteine (SPARC), which are overexpressed in tumor cells and immune cells. The targeted liposomal therapy effectively inhibited the crosstalk between tumor metabolism and immune evasion by suppressing glycolysis and normalizing immune function. This resulted in decreased lactic acid production and angiogenesis, a switch in tumor-associated macrophages (TAMs) to an anti-tumor phenotype, and an improved anti-tumor function of effector CD8+ T cells.

#### 2.2.2. Solid Lipid Nanoparticles and Nanostructured Lipid Carriers

Solid lipid nanoparticles (SLNs) and Nanostructured lipid carriers (NLCs) are appealing options for delivering drug substances because they are easy to produce, compatible with biological systems, capable of breaking down naturally, and can be produced on a large scale [57,58].

In a study, Lf-conjugated PTX-loaded SLNs were developed to treat lung cancer with reduced side effects [59]. The in vitro results showed that the Lf-conjugated nanoparticles were more cytotoxic on BEAS-2B human bronchial epithelial cells and accumulated more in the lungs in rats than unconjugated nanoparticles and free PTX.

To selectively deliver DTX to the brain tissue, DTX-loaded SLNs were prepared and then coated with Lf [60]. Compared to the free drug, the coated SLNs exhibited significantly higher toxicity and apoptotic effect in U87 MG cells, resulting from the higher uptake in this cell line. In another approach, SLNs modified with tamoxifen (TX), which can reverse MDR [61], and Lf were investigated as the carriers for carmustine (NCNU) [62]. The dual-modified TX-Lf-BCNU-SLNs demonstrated 10-fold higher BBB permeability of BCNU. These SLNs also demonstrated higher cellular internalization into U87 MG cells, thereby leading to higher cytotoxicity compared to the free drug. Another type of dual-targeting SLNs was fabricated using Lf and wheat germ agglutinin (WGA) to deliver etoposide for GBM treatment [63]. The formulation showed 5.6-fold higher etoposide permeability to cross the BBB and enhanced the antiproliferative activity in U87MG cells.

A combination therapy for GBM was fabricated by co-encapsulating TMZ and vincristine into NLCs co-conjugated with Lf and RGD peptide [64]. In vitro studies showed that the co-conjugated NLCs enhanced uptake into U87 MG cells compared to unconjugated or single-conjugated NLCs, resulting in synergistic cytotoxicity. In addition, in vivo results revealed significant drug accumulation in tumors and markedly higher tumor suppression in mice bearing U87 MG cells treated with co-modified nanoparticles.

### 2.3. Polymer-Based Nanoparticles Modified or Loaded with Lf

#### 2.3.1. Natural Polymer-Based Nanoparticles

As a biocompatible, biodegradable, and nontoxic material with easy preparation, chitosan has received much attention as a drug delivery system to enhance drug solubility and stability, improve efficacy, and lower toxicity [65,66,67]. In a study, Abu-Serie et al. formulated Lf-coated chitosan NPs encapsulated with lactoperoxidase (LPO) [68]. The Lf-coated, LPO-loaded NPs improved the cytotoxic effect and enhanced the apoptotic effect on HepG-2, Caco-2, PC-3, and MCF-7 cells compared to noncoated NPs or 5-FU, while sparing normal cells.

Recently, sodium alginate was used to crosslink with Lf to form core–shell nanohybrids to deliver a combination of three drugs, namely pemetrexed (an antimetabolite, honokiol), a phenol extracted from *Magnolia grandiflora*, and rosuvastatin, with high drug loading [69]. The nanohybrid system improved cellular uptake and cytotoxicity in MCF-7 breast cancer cells compared to free drugs and free dual/triple drug combinations. Especially, these nanohybrids reduced tumor size and inhibited the expression levels of VEGF-1 and Ki-67 in mice bearing an Ehrlich Ascites Tumor (EAT).

To target both the blood–brain barrier and brain glioma cells, Su et al. developed a system using bovine serum albumin nanoparticles (BSA-NPs) co-modified with Lf and poly(ethylene glycol) methyl ether 2000 (mPEG2000) for encapsulating DOX [70]. The results showed that the higher the levels of Lf and mPEG2000, the bigger the size and the smaller the zeta potentials of the dual-targeted nanoparticles. In vitro results showed that these nanoparticles exhibited higher uptake and cytotoxicity in C6 glioma cells and brain capillary endothelial cells (BCECs) than all other nanoparticles and/or free DOX. In addition, these nanoparticles remarkably increased drug accumulation in the brain while decreasing the accumulation in the heart and kidney, demonstrating the potential of the dual-targeting system in glioma treatment to achieve reduced systemic toxicity.

Wang and colleagues attempted to remodel the tumor immune microenvironment (TIME) and glucose metabolism by co-delivering two anticancer drugs, namely shikonin (SHK) and JQ1 [71]. SHK suppresses NF-κB-regulated gene products and pyruvate kinase-M2 (PKM2), induces immunogenic cell death, and inhibits cancer glucose metabolism [72,73,74], while JQ1 selectively inhibits the Bromodomain-containing protein 4 (BRD4) signaling pathway [75]. They used a green thermal denaturation method to prepare Mannopyranoside-Lf (Man-Lf) NPs, which target mannose receptors expressed on tumor-associated macrophages (TAMs) to help localize the NPs for subsequent inhibition of lactate production. The authors found that Man-Lf NPs led to increased calreticulin (CRT) expression on the tumor cell membrane, accumulated significantly in tumors, and demonstrated superior anti-tumor efficacy in CT26 tumor-bearing mice compared to other drugs and Lf NPs. Based on these findings, it appears that Man-Lf-NPs have the potential as a targeted therapy for colorectal cancer in human patients.

#### 2.3.2. Synthetic Polymer-Based NPs

**Figure 4:**
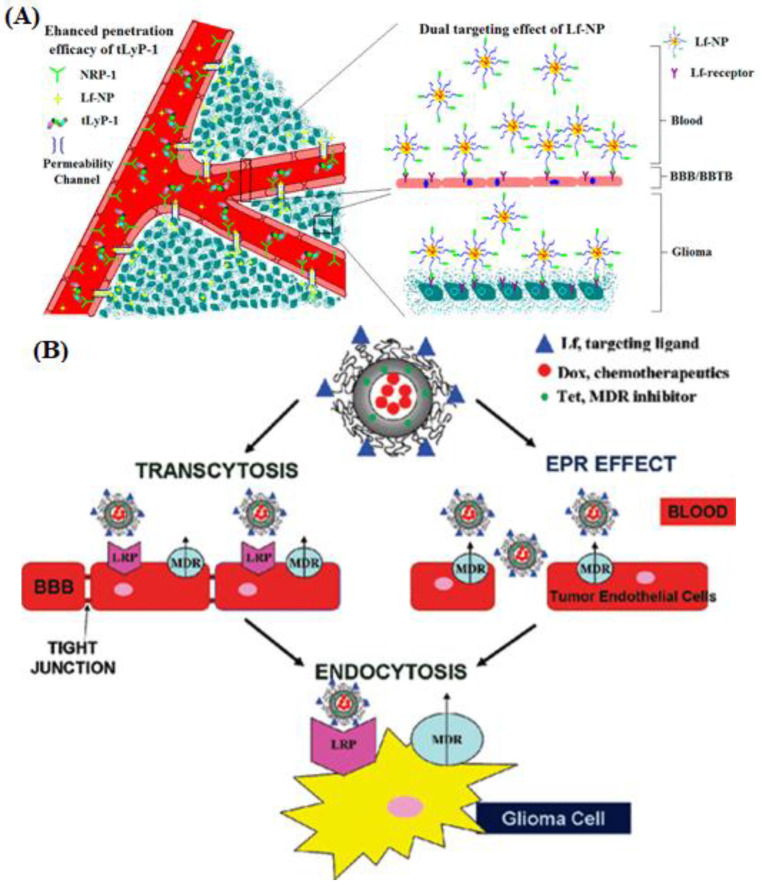
(**A**). Schematic diagram representing the dual targeting effect of Lf-coated PEG-PLA nanoparticles when combined with tLyP-1 peptide. Reprinted with permission from [76]. (**B**). Design of lactoferrin-conjugated biodegradable polymersome for glioma targeting. Reprinted with permission from [81].

A biodegradable system consisting of Lf-conjugated poly(ethylene glycol)–poly(lactide) (PEG–PLA) nanoparticles (NPs) was formulated to improve the transportation of PTX to glioma cells [76]. A tumor-homing peptide, namely tLyP-1, was loaded into the Lf-coated nanoparticles to enhance its accumulation and penetration into the glioma tissue (Figure 4A). Compared to unconjugated NPs, the Lf-loaded nanoparticles exhibited higher cellular uptake into BCEC and C6 cell lines, higher cytotoxicity, and deeper penetration in 3D glioma spheroids. The combination of Lf-coated nanoparticles and tLyP-1 also enhanced tumor targeting, extravasation into the glioma vessels, and prolonged survival of mice bearing intracranial C6 glioma.

A dual-targeting system was created via coupling both Lf and folic acid to the surface of PLGA NPs to enhance the delivery of etoposide across the BBB [77]. As expected, the dual-targeting systems enhanced the permeability across the BBB and cytotoxicity in the U87 MG cell line compared to uncoated PLGA NPs and free etoposide, which may be explained by the fact that it can strongly bind to both LF receptors and folate receptors [78].

An Lf-conjugated nanobubble formulation comprising of an amphiphilic poly(aminoethyl ethylene phosphate)/poly(L-lactide) (PAEEP-PLLA) copolymer was fabricated to load liquid perfluoropentane inside the core [79]. The Lf-coated nanobubbles demonstrated high cellular uptake in rat C6 glioma cells and powerful, long-lasting, and tumor-enhanced ultrasonic contrast ability in vivo, suggesting the potential of these nanobubbles in tumor-targeting ultrasonic imaging as an ultrasonic contrast agent.

The blood–brain barrier (BBB) and multidrug resistance (MDR) are the main reasons for the poor prognosis of glioma patients after chemotherapy [80]. To address these issues, a new drug delivery system, Lf-conjugated biodegradable polymersome (Lf-PO-DOX/TET) composed of methoxy poly(ethylene glycol)-poly(ε-caprolactone) (MPEG3k-PCL15k) and R-carboxyl poly(ethylene glycol)-poly(ε-caprolactone) (HOOC-PEG3.4kPCL15k), was developed to encapsulate both DOX and tetrandrine (TET) (Figure 4B) [81]. This system integrates both BBB-penetrating and glioma-targeting properties, and an MDR inhibitor. Lf-PO-DOX/TET showed stronger cytotoxicity against C6 glioma cells and higher uptake into these cells than all other polymersomes, namely PO-DOX, PO-DOX/TET, and Lf-PO-DOX. In vivo imaging analysis showed that the Lf-PO-DOX/TET group significantly decreased the tumor size and prolonged survival time compared to the other groups.

To synergize the effects of chemo/herbal combinations in breast cancer treatment, Khattab et al. created nanogels using Lf and carboxymethyl cellulose for the delivery of pemetrexed and honokiol [82]. The nanogels were able to effectively deliver both drugs, showing sustained release and greater effectiveness against MDA-MB-231 breast cancer cells than the free drugs. Results from the in vivo studies showed that the nanogels suppressed the expression levels of VEGF-1 and Ki-67 and increased the expression of caspase-3, thereby significantly inhibiting tumor growth in EAT-bearing mice.

Dendrimers are nanoscopic hyperbranched and three-dimensional structures that have been studied for various applications, including drug delivery, due to the presence of functional groups at the surface and interior that can be used to attach drugs and targeting molecules [83,84]. Kurmi et al. aimed to develop a way to target the drug methotrexate (MTX) specifically to the lungs using 5.0G poly(propylene imine) (PPI) dendrimers conjugated with Lf [85]. The researchers found that the Lf-conjugated dendrimers were significantly better than the plain dendrimers or free MTX at targeting the lungs, with 1.5 times and 2.5 times higher accumulation of drugs, respectively.

The absence of safe and intravenous delivery systems for the selective delivery of therapeutic genes to tumors significantly reduces the effectiveness of using genes in cancer treatment [86]. In a study, Dufès tried to explore whether Tf-bearing generation 3-diaminobutyric polypropylenimine dendrimer (Tf-DAB) could result in targeted gene delivery to tumors through intravenous administration to enhance therapeutic outcomes [87]. Once administered intravenously, gene expression was primarily found in the tumors. As a result, administering the delivery system combined with plasmid DNA encoding tumor necrosis factor alpha (TNFα) resulted in rapid and sustained tumor regression for 40 days, during which all A431 tumor-bearing mice survived (90% of which had a complete response) and were well-tolerated. In more recent research, this author’s group also used Tf-DAB to co-deliver TNFα, a tumor-necrosis factor related apoptosis-inducing ligand (TRAIL), and interleukine (IL)-12 genes [88]. They found that the co-delivered dendriplexes significantly suppressed tumor growth in mice bearing DU145 or PC-3 prostate cancer with no apparent side effects. Therefore, Tf-bearing polypropylenimine dendrimers may be a promising method for delivering genes for cancer therapy.

The application of Lf as the targeting moiety is broad across a variety of material bases as summarized in Table 1. 

### 2.4. Drug–Lf Conjugates

As a naturally hydrophilic protein, lactoferrin can be directly conjugated to other drug compounds in order to enhance their solubility, reduce their systematic side effects, and improve targeted delivery through the use of Lf receptors, which are overexpressed on numerous cell types [89].

In an attempt to target colon cancer, curcumin was conjugated to Lf [90]. The conjugated CUR significantly improved aqueous solubility, cellular uptake, and cytotoxicity in HCT116 human colon cancer cells compared to free curcumin. In another study, a conjugate of Lf and DOX was prepared to investigate its potential application in prostate cancer [91]. The synthesized conjugate exhibited higher uptake into DU145 prostate cancer cells, resulting in four-fold higher cytotoxicity in this cell line than free DOX. Additionally, the orally administered conjugate demonstrated superior anti-tumor efficacy, with a safer systemic toxicity profile in transgenic mice exhibiting adenocarcinoma of the prostate (TRAMP).

In order to synergize the anticancer efficacy of two hydrophobic drugs, namely celastrol and DTX, in breast cancer treatment, these two drugs were attached to Lf [92]. The resulting conjugate self-assembled into stable nanoparticles with high drug loading. In comparison to the free drug combination, the nanoconjugate significantly reduced tumor volume, prolonged survival rate, and remarkably suppressed expression levels of NF-κB p65, COX-2, TNF-α, and Ki-67 in mice bearing an Ehrlich Ascites Tumor (EAT). Another approach to tackle breast cancer was based on the combined chemo/hormonal therapy between MTX and the aromatase inhibitor exemestane [93]. In this system, exemestane was encapsulated into liquid crystalline nanoparticles, followed by electrostatic coating with the cationic Lf-MTX conjugate. In vitro studies showed that the dual drug-loaded nanoparticles exhibited higher cellular internalization into MCF-7 breast cancer cells than non-targeted liquid crystalline nanoparticles and demonstrated significantly higher cytotoxicity in this cell line than the free drug combination.

When used as an anticancer agent over the long term, heparin can induce several side effects, such as local breeding, pulmonary embolism, and thrombocytopenia [94,95,96]. In order to overcome these limitations, a novel orally absorbable form of heparin was developed by conjugating Lf to heparin via amide linkage [97]. The Lf-heparin conjugate was stable in acidic environments and exhibited a 4.4-fold higher circulation time than that of the intravenous injection and demonstrated an antiangiogenic effect in vitro without toxicity. Following oral administration, the conjugate was absorbed in the small intestine and transported specifically to the tumor sites, thereby inhibiting the progression of angiogenesis in glioblastoma orthotopic mice.

**Table 1:** Summary of nanoparticles using Lf as a targeting moiety.

Material Base	Carriers	Anti-Cancer Dg	Results	Ref.
1. Inorganic NPs				
1.1. Silica NPs	Ultra-small size with large pore silica NPs (USLPs)	Doxorubicin (DOX)	Enhanced BBB permeation, enhanced internalization and apoptosis in U87 cells	[18]
		Temozolomide (TMZ)	Improved BBB permeation and apoptosis	[19]
	Mesoporous silica nanoparticles (MSNs)	Pemetrexed (PMT) and ellagic acid (EA)	Enhanced cellular uptake and cytotoxicity in MCF-7 breast cancer cells	[23]
1.2. Magnetic NPs	Maghemite and magnetite NPs	–	Enhanced cytotoxicity in 4T1 breast cancer cells	[27]
	Mesoporous maghemite nanoparticles	DOX	Improved anti-cancer efficacy in vitro and in vivo	[28]
	Superparamagnetic iron oxide nanoparticles; graphene oxide (GO) sheets	DOX	Improved intracellular uptake and cytotoxicity to C6 glioma cells	[29]
	Superparamagnetic iron oxide (SPIO) nanoparticles; 10,12-pentacosadiynoic acid (PCDA)	Curcumin (CUR)	Enhanced CUR accumulation in brain and inhibited tumors in vivo	[30]
	Iron oxide nanoparticles, polyacrylic acid (PAA) and polyvinyl alcohol (PVA)	CUR and DOX	Enhanced cytotoxicity in RG2 glioma cells and reduced tumor growth	[31]
	Mesoporous iron oxide nanoparticles (MIONs)	Perfluorohexane (PFH) and paclitaxel (PTX)	Enhanced drug accumulation in tumor and and anti-cancer efficacy in vitro and in vivo	[32]
	YOF: Nd^3+^-MnO_2_ core–shell nanoparticles	Indocyanine green and glucose oxidase	Exhibited synergistic effect of starvation/photodynamic therapy to enhance anticancer efficacy in vitro	[36]
	Hollow mesoporous copper sulfide nanoparticles	TMZ and glucose oxidase	Exhibited synergistic chemo/chemodynamic/photothermal/starvation therapy to enhance anticancer efficacy in vitro and in vivo	[37]
2. Lipid-based NPs				
2.1. Liposomes	Liposomes	DOX	Enhanced BBB permeability and cellular uptake	[41,42]
	Liposomes coated with RGD peptide	Docetaxel (DTX)	Enhanced accumulation in gliomas and improved mice survival	[43]
	Liposome coated with muscone	DTX	Enhanced cellular internalization and anticancer efficacy in vitro and in vivo	[46]
	PEGylated liposomes	DOX	Enhanced uptake and cytotoxicity in ASGP-R positive cells; improved tumor inhibition in vivo	[49,50]
	PEGylated liposomes	DOX	Increased cellular uptake and tumor accumulation; enhanced tumor radiochemotherapy in vivo	[51]
	Liposomes	Panobinostat and JQ1	Activated anti-tumor immunity responses and inhibited tumor growth and metastasis	[54]
2.2. SLNs and NLCs	SLNs	Paclitacel (PTX)	Enhanced cytotoxicity in BEAS-2B human bronchial epithelial cells and drug accumulation in rats’ lungs	[59]
	SLNs	DTX	Enhanced cellular uptake, cytotoxicity, and apoptosis in U87 MG cells	[60]
	SLNs	Tamoxifen and carmustine	Enhanced cellular uptake and cytotoxicity in U87 MG cells	[62]
	SLNs modified with wheat germ agglutinin (WGA)	Etoposide	Enhanced BBB permeation and antiproliferative activity in U87 MG cells	[63]
	NLCs modified with RGD peptide	TMZ	Enhanced cellular uptake and cytotoxicity in U87 MG cells; improved tumor inhibition in vivo	[64]
3. Polymer-based NPs				
3.1. Natural polymer	Chitosan NPs	lactoperoxidase (LPO)	Improved cytotoxicity and apoptosis in HepG-2, Caco-2, PC-3, and MCF-7 cells	[68]
	Sodium alginate	Pemetrexed, honokiol, and rosuvastatin	Enhanced cellular uptake into MCF-7 breast cancer cells and improved anticancer efficacy in vitro and in vivo	[69]
	Bovine serum albumin NPs modified with mPEG2000	DOX	Enhanced cellular uptake and cytotoxicity in C6 glioma cells	[70]
	Mannopyranoside	Shikonin (SHK) and JQ1	Enhanced accumulation in tumors and anticancer efficacy in vivo	[71]
3.2. Synthetic NPs	PEG–PLA NPs	PTX	Enhanced cellular uptake and cytotoxicity in C6 glioma cells	[76]
	PLGA NPs	Etoposide	Enhanced BBB permeation and cytotoxicity in U87 MG cells	[77]
	PAEEP-PLLA NPs	Perfluoropentane	Enhanced cellular uptake in C6 glioma cells; exhibited strong, long-lasting, and tumor-enhanced ultrasonic contrast ability in vivo	[79]
	Biodegradable polymersomes	DOX and tetrandrine (TET)	Enhanced cellular uptake and cytotoxicity in C6 glioma cells and improved anticancer efficacy in vivo	[81]
	Carboxymethyl cellulose	Pemetrexed and honokiol	Enhanced anticancer efficacy in vitro and in vivo	[82]
	5.0G poly(propylene imine) (PPI) dendrimers	Methotrexate (MTX)	Enhanced drug accumulation in lungs	[85]
	Generation 3-diaminobutyric polypropylenimine dendrimers	Plasmid DNA encoding tumor necrosis factor alpha (TNFα)	Improved anticancer efficacy in vivo	[87]
		TNFα, tumor-necrosis factor related apoptosis-inducing ligand (TRAIL), and interleukine (IL)-12 genes	Improved anticancer efficacy in vivo with no side effects	[88]
4. Drug-Lf Conjugates	–	CUR	Improved aqueous solubility, cellular uptake, and cytotoxicity in HCT116 human colon cancer cells	[90]
	–	DOX	Enhanced uptake and cytotoxicity in DU145 prostate cancer cells; improved tumor inhibition with lower systemic toxicity in mice	[91]
	–	Celastrol and DTX	Suppressed tumor growth and prolonged survival in EAT-bearing mice	[92]
	–	MTX and exemestane	Enhanced cellular uptake and cytotoxicity in MCF-7 breast cancer cells	[93]
	–	Heparin	Improved circulation time and exhibited antiangiogenic activity in vitro and in vivo	[97]

## 3. Nanoparticles Carrying Lf as an Active Agent

The activities of Lf in cancer management were reported elsewhere [5,98], but the studies that recruit Lf as the active agent were still limited. That could be the complexity of the structural molecules or the high dose requirement.

Among a few efforts that are presented in Table 2, Abu-Serie et al. loaded bovine Lf into chitosan nanoparticles [68]. Studies on the Caco-2, HepG-2, MCF-7, and PC-3 cells showed the synergistic cytotoxicity effect of bovine Lf and lactoperoxidase (LPO) when LPO was coated outside the nanoparticles. Intriguingly, this anticancer activity is apoptosis-dependent, and the normal cells are out of toxicity. The same scientific group went further on this combination (LPO and Lf) by using Cu and Fe nanometals [99]. At the size range of 21 nm, the combination of LPO-Cu NPs and Lf-Fe NPs enhanced the efficacy by reducing half of the concentration to obtain similar results. The apoptotic (p53 and Bcl-2, Ki-67) and cell cycle (G0 population) pathway was found to be the main target for this therapeutic treatment.

**Figure 5:**
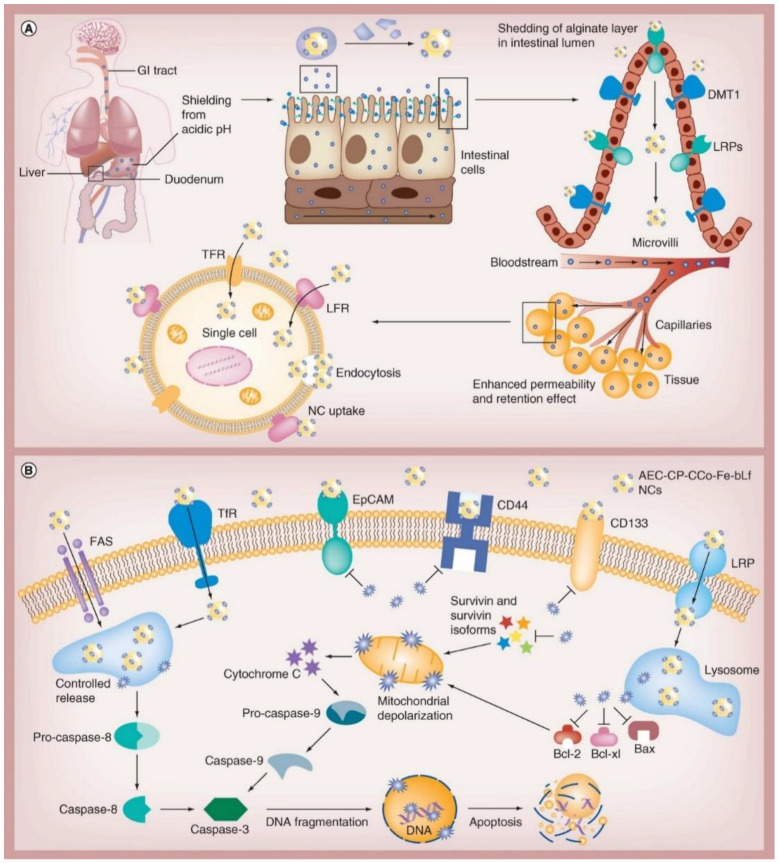
Mechanism involved in internalization of alginate-enclosed chitosan-conjugated calcium phosphate, iron-saturated bovine lactoferrin nanocarriers/nanocapsules (**A**), and their anticancer efficacy (**B**). Reprinted with permission from [101].

To down-regulate survivin, which can promote cancer survival [100], Kanwar et al. encapsulated iron-saturated bovine Lf into nanocapsules developed from chitosan and alginate (Figure 5) [101]. These nanocapsules enhanced cytotoxicity on CaCo-2 cells and reduced cancer stem cell markers in survivin, triple-positive CD133, and CD44 cancer stem-like cells. Interestingly, there were no developments of tumors and toxicity in mice bearing xenograft colon cancer models.

From another aspect, nanoparticles such as chitosan nanoparticles could work as the carriers to identify the location of Lf-induced activity, in which cytoplasmic-targeted Lf nanoparticles at 300 µg/mL could reduce cell viability, whereas nuclear-targeted Lf nanoparticles increase cell viability [102]. This contradiction requires more investigation before Lf could be used for specific indication.

In the world of oncoproteins, some research groups found an innovative way to develop the novel Lf-active agent. Fang et al. prepared the bovine lactoferrin–oleic acid complex that is obtained via van der Waals forces and hydrogen bonds [103]. This complex could kill the tumor cells via a diversity of apoptosis pathways at low concentrations with a LD_50_ of 4.62, 4.88, and 4.95 μM for MCF-7, HepG2, and HT29 cells, respectively. Recently, Elizarova et al. studied this complex in the in vivo model using human Lf [104]. After 48 days of tumor inoculation, none of the mice in the control group survived, whereas the complex saved 70% of mice. Importantly, at the end of the experiment, one-fifth of the mice treated with the complex showed no sight of tumors.

**Table 2:** Summary of nanoparticles encapsulating Lf as an active agent.

Material Base	Carriers	Results	Ref.
Inorganic NPs	Fe NPs	Enhanced cytotoxicity when combined with lactoperoxidase-loaded Cu NPs	[99]
Polymer-based NPs	Chitosan NPs coated with lactoperoxidase	Enhanced cytotoxicity in Caco-2, HepG-2, MCF-7, and PC-3 cells	[68]
	Chitosan/alginate nanocapsules	Decreased viability of CaCo-2 cells and cancer stem cell markers in survivin, triple-positive CD133, and CD44 cancer stem-like cells; treated tumors developed in mice	[101]
	Chitosan NPs	The cytototoxicity depends on the locations targeted	[102]
Complex	Bovine lactoferrin–oleic acid complex	Demonstrated anticancer effect in MCF-7, HepG2, and HT29 cells	[103]
	Human lactoferrin–oleic acid complex	Enhanced tumor inhibition in vivo with higher safety	[104]

## 4. Future Perspectives and Limitations

In the world of cancer management, Lf-integrated nanosystems have been developed, but they are still underdeveloped from a clinical perspective. Most works are fundamental studies in which the targeting properties are dominant. As of now, in the database of clinicals.gov with the keywords “lactoferrin” and “cancer”, only 14 results are reported in which Lf was evaluated for the potential treatment of renal cell carcinoma, hepatocellular carcinoma, non-small cell lung cancer, and squamous cell carcinoma. The positive results seem to need more time to be fully utilized, meaning that a long way is still ahead until we reach the light at the end of the tunnel. Hence, alternatives such as supplements could be reasonable and applicable.

Regarding delivery carriers, Lf should be treated as a biologic with all aspects of stability consideration. To address this issue, the Williams III group has tried thin-film freeze-drying and spray freeze-drying methods to avoid denaturation. Only 2–10 percent of denatured proteins were noticed, suggesting the promise of these approaches. The encapsulation of Lf into the nanocarriers is possibly better in this line [105].

Moreover, local delivery is also promising since it can minimize the risk from external impacts such as heat or moisture. Several works developed the dry powder inhalers of Lf, showing the capability of Lf in managing other conditions such as cystic fibrosis [106], lung injury and fibrosis [107], and infection [108]. These advantages should also shift the paradigm of formulation preparations.

The potential of lactoferrin has been exploited mostly for nutrition and targeting purposes in cancer treatment. The main limitation might be considered as the co-expression of receptors on cancer cells and normal cells, which leads to a risk of toxicity when the targeting strategy is applied. Moreover, when Lf is used as the active agent, the stability and high number of doses required will be critical challenges.

## 5. Conclusions

In conclusion, Lf is a broadly biological molecule that could influence a variety of diseases as well as bind to a wide range of receptors on disease-bearing cells. Different strategies were developed to deliver Lf or to recruit Lf as the target-directed ligand for various drug delivery systems. Whether based on inorganic or organic materials, the investigated nanoparticles contributed greatly to the efficacy of cancer treatment. Some novel approaches such as a complex with oleic acid or the development of local delivery carrier were also discussed. Last but most importantly, the multiple applications in the field of cancer therapy and nanomedicine require more effort to realize their potential in the clinical setting.

## Data Availability

No datasets were generated or analyzed during the current study.
